# A therapeutic leap: how myosin inhibitors moved from cardiac interventions to skeletal muscle myopathy solutions

**DOI:** 10.1172/JCI179958

**Published:** 2024-05-01

**Authors:** Julius Bogomolovas, Ju Chen

**Affiliations:** Department of Medicine, UCSD, La Jolla, California, USA.

## Abstract

The myosin inhibitor mavacamten has transformed the management of obstructive hypertrophic cardiomyopathy (HCM) by targeting myosin ATPase activity to mitigate cardiac hypercontractility. This therapeutic mechanism has proven effective for patients with HCM independent of having a primary gene mutation in myosin. In this issue of the *JCI*, Buvoli et al. report that muscle hypercontractility is a mechanism of pathogenesis underlying muscle dysfunction in Laing distal myopathy, a disorder characterized by mutations altering the rod domain of β myosin heavy chain. The authors performed detailed physiological, molecular, and biomechanical analyses and demonstrated that myosin ATPase inhibition can correct a large extent of muscle abnormalities. The findings offer a therapeutic avenue for Laing distal myopathy and potentially other myopathies. This Commentary underscores the importance of reevaluating myosin activity’s role across myopathies in general for the potential development of targeted myosin inhibitors to treat skeletal muscle disorders.

## Targeting hypercontractility with myosin ATPase inhibitor therapy

In recent years, the treatment of obstructive hypertrophic cardiomyopathy (HCM) has been revolutionized by myosin inhibitors. Over a thousand mutations, predominantly in genes encoding sarcomeric proteins responsible for generating and regulating cardiac contraction, have been identified as causative for HCM (1). *MYH7* encodes β myosin heavy chain (βMyHC), and missense mutations in the motor domain–containing head region lead to HCM (1). Functionally, many HCM mutations lead to cardiac hypercontractility, and this hypercontractilty can lead to obstruction due to cardiac septal thickening (2). Now in clinical use, mavacamten, formerly known as MYK-461, is a small-molecule allosteric and reversible inhibitor of myosin ATPase activity that directly targets cardiac hypercontractility, providing therapeutic benefits for patients with obstructive HCM. Preclinical studies in genetic HCM mouse models, carrying human disease–causing mutations in the myosin head where ATPase activity is located, demonstrated that early administration of mavacamten can reduce the development and progression of HCM’s characteristic morphological, histopathological, and molecular alterations (3). Although the molecular target and binding site for mavacamten is the myosin head, data from the phase III EXPLORER-HCM clinical trial (4) showed that mavacamten is beneficial even in HCM patients without sarcomere gene variants, indicating that myosin ATPase activity inhibition is beneficial even when there are other causes of cardiac hypercontractility and outflow obstruction. This observation is supported by experimental data showing that mavacamten can reduce pathological hypercontractility caused by mutations in other sarcomeric components (5–9). The success of a myosin inhibitor in treating obstructive HCM demonstrates that targeted inhibition of myosin ATPase is an effective approach for HCM therapy. Clinical and experimental data suggest that myosin ATPase inhibition has more extensive effects and therapeutic potential than initially thought.

## Tackling myosin in Laing distal myopathy

In this issue of the *JCI*, Buvoli et al. report on therapeutic effects of myosin ATPase inhibition in Laing distal myopathy, a specific form of skeletal myopathy characterized by mutations in the *MYH7* gene, which, in addition to its cardiac expression, is also found in type I slow skeletal muscle fibers (10). The authors concentrated their efforts on the R1500P variant, which, like most Laing distal myopathy mutations, is located within the coiled-coil rod domain of βMyHC. Buvoli and colleagues generated a transgenic mouse model expressing WT and R1500P-mutant βMyHC under control of the muscle creatine kinase (MCK) promoter. The researchers opted for a transgenic approach over a knock-in strategy, given the naturally low abundance of type I fibers in mice, necessitating overexpression of βMyHC to adequately model the disease phenotype found in human skeletal muscle. The inclusion of the WT βMyHC control was a deliberate choice to account for the differences in type I fiber proportions, ensuring that any observed effects could be accurately attributed to the R1500P mutation rather than variations in muscle fiber composition due to overexpression of fiber type I–associated βMyHC. The phenotype observed in these mice mirrored several key features of Laing distal myopathy, including muscle weakness and some aspects of muscle histopathology. Notably, in an ex vivo experimental setup, mice expressing the R1500P mutation exhibited muscle hypercontractility and enhanced fatigue. Moreover, biochemical measurements in isolated myofibrils revealed an elevated ATP turnover rate in mutant myofibrils from mouse models and patients, indicating increased myosin ATPase activity due to a shift toward a more energy-consuming disordered-relaxed (DRX) state of the myosin heads. This critical observation provided the rationale for testing the mavacamten analog MYK-581 in human muscle biopsies as well as in mouse models.

**Figure 1:**
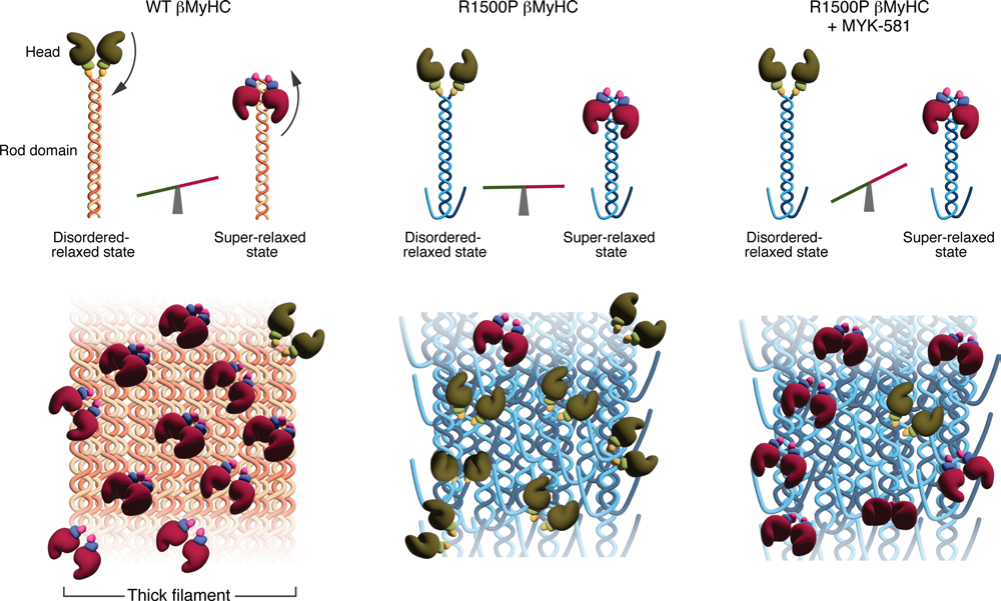
The myosin inhibitor MYK-581 corrects the molecular phenotype of R1500P myofibrils. The equilibrium between the DRX state and the SRX state of βMyHC within the sarcomere favors the SRX state in healthy myofibrils, indicating that more WT βMyHC molecules are present in the SRX state. In contrast, R1500P βMyHC mutations cause the distribution of myosin heads to skew toward the DRX state. While R1500P βMyHC demonstrates near-equal distribution between DRX and SRX states, reflecting the pathological condition of Laing distal myopathy, treatment of R1500P βMyHC myosin–expressing myofibrils with MYK-581 causes a substantial shift toward the SRX state, highlighting the drug’s ability to restore a healthy balance between myosin head states. Notably, the structural effects of the R1500P mutation on the thick filament structure persist despite the shift toward more SRX myosin heads induced by MYK-581 treatment, and the distorted organization of the thick filament caused by the R1500P mutation remains.

Mavacamten’s therapeutic efficacy in HCM is attributed to its ability to shift myosin heads from the DRX state to the super-relaxed (SRX) state, thereby reducing ATP consumption and improving cardiac function (8). Buvoli and co-authors successfully confirmed this mechanism of action, demonstrating that the shift of the myosin heads toward the SRX state, induced by the oral administration of MYK-581, improved muscle function in vivo, thereby addressing the underlying molecular dysfunction observed in the disease (Figure 1). Notably the therapeutic effect of MYK-581 was demonstrated by the restoration of muscle endurance and running capacity in the R1500P mice, observed as early as four days after treatment using the voluntary wheel-running paradigm. Finally, the authors introduced a Markov model that simulates mouse activity patterns and performed a detailed analysis of running statistics, showing that MYK-581 enhanced the endurance and activity of the R1500P mice by modulating running and resting times.

Buvoli et al. illuminate a fascinating aspect: muscle hypercontractility previously associated exclusively with the pathogenesis of HCM actually plays an important role in the development of skeletal myopathy. Clinical data have demonstrated that mavacamten’s ability to shift myosin heads from the DRX state to the SRX state offers clinical benefits not only for patients with HCM who carry mutations in the myosin ATPase domains but also for those with other underlying causes. Buvoli and authors demonstrate that the same pathomechanism and treatment approach can be extended to Laing distal myopathy. The question then arises: what mechanism underlies this phenomenon in skeletal myopathies? Through x-ray diffraction experiments on myofibrils, the authors discovered a distortion in the periodic arrangement of myosin within the myofibril. They speculated that the accommodation of a proline in the myosin coiled-coil altered the thick and thin filament superstructure and register, leading to rogue myosin heads that were more likely to remain in the DRX state. The authors hypothesized that the effects of the R1500P mutation in the context of thick filament are intermolecular: the warping of the myosin coiled-coil domain introduced by the proline mutation diffuses across the sarcomere by the staggered interaction between myosin molecules. As a result, SRX-stabilizing interactions occurring in the thick filament backbone become disrupted (Figure 1). The proposed concept was further supported by enhanced cross-bridge relaxation observed in isolated mutant myofibrils.

## Beyond Laing distal myopathy

The results of this study open avenues for exploring skeletal myopathies from a fresh perspective. Notably, mutations located in the C-terminal region of the MyHC rod domain are causative not only for Laing distal myopathy but also for myosin storage myopathies, some of which have missense mutations resulting in a change to proline (11). Although mutations that cause myosin storage myopathy impair the proper integration of myosin into the sarcomere, leading to the extrasacromeric aggregates that give the condition its name, those mutants that do integrate into the thick filament introduce a structural disruption akin to the R1500P mutation seen in Laing distal myopathy. Consequently, such structural disruptions could lead to pathological changes within the sarcomere similar to those described in Buvoli et al. for Laing distal myopathy. Additionally, this study encourages a reevaluation of non-myosin skeletal myopathies, such as nemaline myopathy, which shares disruption of the myosin SRX state with Laing distal myopathy (12). Given that myosin inhibition has been beneficial in HCM mutations caused by thin filament mutations, this study warrants a broader exploration of myosin activity in skeletal myopathies caused by thin filament mutations. Furthermore, given that existing myosin inhibitors are tailored for cardiac myosins, the development of inhibitors specifically targeting skeletal myosins presents opportunities for the treatment of skeletal myopathies caused by mutations exclusively in skeletal myosins. This approach gains further validation from ongoing studies on fast myosin inhibitors in the context of Duchenne muscular dystrophy, in which inhibition of fast skeletal myosin activity has shown potential therapeutic benefits (13). Additionally, this strategy is supported by cases like MYH2 myopathies, which are characterized by increased basal myosin ATP consumption and a reduced SRX state in vivo (14), reinforcing the promise of myosin inhibition as a therapeutic strategy for a broad spectrum of skeletal myopathies.

## Version 1 05/01/2024

Electronic publication
